# Editorial: Biomarkers in Drug Hypersensitivity

**DOI:** 10.3389/fphar.2017.00348

**Published:** 2017-06-07

**Authors:** José A. G. Agúndez, Silvia Selinski, Emanuela Corsini, Klaus Golka, Elena García-Martín

**Affiliations:** ^1^Department of Pharmacology, University of ExtremaduraCáceres, Spain; ^2^Leibniz Research Centre for Working Environment and Human Factors at Technische Universität Dortmund (IfADo)Dortmund, Germany; ^3^Department of Environmental Science and Policy, Università degli Studi di MilanoMilan, Italy

**Keywords:** ADRs, drug hypersensitivity, drug allergy, HLA genes, DILI

This special issue on biomarkers in drug hypersensitivity consists of 11 articles focusing on recent advancements related to this exciting field. Biomarkers, especially those based on pharmacogenomics testing, have proved to be extremely useful for type A adverse drug reactions (ADRs). Clinical practice guidelines based on biomarker testing are presently being developed and updated for type A ADRs (see, for instance the Clinical Pharmacogenetics Implementation Consortium website https://cpicpgx.org/guidelines/).

The World Health Organization defines ADRs as any noxious, unintended, and undesired effect of a drug that occurs at doses used for prevention, diagnosis, or treatment. Major ADR categories include type A—predictable reactions (about 80% of all ADRs), and type B—unpredictable, reactions. Where predictable reactions are usually dose dependent, related to the known pharmacologic actions of the drug, and occur in otherwise healthy subjects, unpredictable reactions are generally dose independent, unrelated to the pharmacologic actions of the drugs, and occur only in susceptible subjects. Compared to type A, type B ADRs are extremely complex and these include drug intolerance, drug idiosyncrasy, drug allergy, and pseudo-allergic reactions.

Böhm and Cascorbi elegantly summarized the different reaction types, mechanisms and known biomarkers for type B ADRs. Among these, the HLA-B alleles are highly relevant for delayed T-cell mediated reactions with abacavir (Martin et al., 2014) and carbamazepine (Leckband et al., 2013). In another article included in this Research Topic Sukasem et al. confirmed the relevance of HLA-B in the occurrence of adverse reactions (e.g., Stevens-Johnson syndrome and toxic epidermal necrolysis) secondary to the use of allopurinol in Thai patients. A previously published CPIC guideline contraindicated the use of allopurinol for carriers of HLA-B^*^5801 (Hershfield et al., 2013), although it should be borne in mind that the Thai population displays an unusually high frequency for carriers of such variant alleles and therefore, it is crucial to measure the strength of the association in this population in order to gain more ground on the clinical implementation of preemptive pharmacogenomics tests for HLA-B alleles. In spite of the utility of HLA testing, clinical implementation is hampered due to its technical complexity and because of the potential source of heterogeneity related to the use of diverse genotyping procedures (revised in Martin et al., 2012). The contribution by Chua and Ng in this Research Topic analyzes the potential of an additional procedure for HLA testing based on nanopore sequencing mechanisms, seeking a rapid and useful tool for the detection of genetic markers for drug hypersensitivity. They concluded that the procedure is promising, although there is still room for improvement.

Besides genetic biomarkers, clinical and analytical biomarkers provide crucial information. A careful assessment of clinical phenotypes, for instance, is essential to improve the accuracy of biomarkers as predictors of disease evolution and/or therapy response. Pérez-Alzate et al. identified an unusual clinical presentation among patients who were selective responders to paracetamol or a single NSAID, which usually present with urticaria and/or angioedema and anaphylaxis: A subgroup of selective responders presented with rhinitis and/or asthma with no skin manifestation. These findings suggest the occurrence of a new clinical phenotype for selective responders to NSAIDs. A particularly severe phenotype of drug-induced hypersensitivity is drug-induced liver injury. Robles-Díaz et al. summarized current knowledge on biomarkers related to diagnosis, phenotypes, clinical course and prognosis. Among these, mechanistic-based biomarkers such as the proteins High-mobility group box 1 and Keratin-18, or the micro-RNA miR-122 hold great promise.

Drug-induced hypersensitivity is often related to metabolic activation and haptenization of drug metabolites. Hence, factors that influence the pharmacokinetics of drug and metabolites may contribute to the development of some drug-induced hypersensitivity reactions. This implies that processes such as biotransformation and excretion, which are typically involved in type A adverse drug reactions, may have a role in hypersensitivity reactions too. Nuin et al. demonstrated that the active trifusal metabolite 2-hydroxy-4-trifluoromethylbenzoic acid, which causes photoallergy, is covalently bound to a protein model after photoactivation.

Other clinical presentations of drug-induced hypersensitivity reactions correspond to non-allergic mechanisms, usually associated with the release of inflammatory transmitters. Of these, eicosanoids play a prominent role in inflammation and are thought to be involved in cross hypersensitivity to NSAIDs. In this regard, it has been shown that some genetic variants of the arachidonic acid pathway influence the risk of developing such cross-hypersensitivity reactions (Cornejo-Garcia et al., 2012). Cornejo-García et al. analyzed the genetic variability of prostaglandin and leukotriene receptors, seeking for genetic biomarkers which alone, or combined with polymorphisms of the genes coding for the cyclooxygenase enzymes (Agundez et al., 2014, 2015), may help in the understanding of the mechanisms underlying cross-hypersensitivity to NSAIDs.

Several signal transduction pathways participate and modulate the development and the clinical presentation of drug hypersensitivity once the reaction is triggered. One of these depends on the interplay of IgE response and the consequent release of mediators. Amo et al. studied the genetic variability of the high-affinity IgE receptor (Fcε RI) and the variability in genes coding for enzymes involved in histamine homeostasis in patients with selective hypersensitivity to NSAIDs. They concluded that polymorphisms in the diamine oxidase (DAO) gene that have functional consequences (Ayuso et al., 2007) are involved in the clinical presentation of these selective hypersensitivity reactions, as has been reported previously in patients with cross hypersensitivity to NSAIDs (Agundez et al., 2012). Sánchez-Gómez et al. reviewed the role of enzymes involved in the generation of danger or co-stimulatory signals, such as GSTP1-1 and aldose reductase, in drug hypersensitivity. These enzymes are important regulators of the balance of inflammatory mediators, they participate in allergic processes, they can metabolize drugs and they are covalently modified by drugs, thus indicating a high potential for these enzymes in future research on mechanisms underlying drug hypersensitivity.

Two papers in this research topic focused on *in vitro* models. Galbiati et al. analyzed the potential of the use of THP-1 cell lines and interleukin-8 production together with CD86 and CD54 expression for pre-clinical immune safety evaluation studies. The proposed *in vitro* method benefits from a rationalistic approach with the idea that allergenic drugs share with chemical allergens common mechanisms of cell activation. In addition, they described the experimental conditions and markers to identify drug sensitizers, also assessing the state of the art of *in vitro* models to assess the allergenic potential of drugs. Steiner et al. reviewed the literature on a different model used in clinical studies, the Basophil Activation Test (BAT). After analyzing the potential of this procedure in hypersensitivity to beta-lactams, quinolones, neuro-muscular blocking agents, contrast media, chemotherapeutics, and NSAIDs, among other drugs, they concluded that BAT constitutes a safe, complement of *in vivo* tests in immediate drug hypersensitivity reactions.

We would like to thank all the contributors whose valuable work has helped us to present wide-ranged aspects in this field.

## Author contributions

All authors listed, have made substantial, direct and intellectual contribution to the work, and approved it for publication.

### Conflict of interest statement

The authors declare that the research was conducted in the absence of any commercial or financial relationships that could be construed as a potential conflict of interest.
